# Estradiol Replacement Improves High-Fat Diet-Induced Obesity by Suppressing the Action of Ghrelin in Ovariectomized Rats

**DOI:** 10.3390/nu12040907

**Published:** 2020-03-26

**Authors:** Naoko Yokota-Nakagi, Haruka Takahashi, Mizuho Kawakami, Akira Takamata, Yuki Uchida, Keiko Morimoto

**Affiliations:** 1 Department of Environmental Health, Faculty of Human Life and Environment, Nara Women’s University, Kitauoya Nishimachi, Nara 630-8506, Japan; oan_yokota@cc.nara-wu.ac.jp (N.Y.-N.); takahashih@jfrl.or.jp (H.T.); kawakami-m@sato-yakuhin.co.jp (M.K.); takamata@cc.nara-wu.ac.jp (A.T.); yukioto@cc.nara-wu.ac.jp (Y.U.); 2 Department of Health and Nutrition, Faculty of Health Science, Kyoto Koka Women’s University, 38 Kadono-cho, Nishikyogoku, Ukyo-ku, Kyoto 615-0882, Japan

**Keywords:** estradiol replacement, ghrelin, growth hormone secretagogue receptor, energy intake, high-fat diet-induced obesity, ovariectomized rat

## Abstract

This study aims to investigate the effects of estradiol replacement on the orexigenic action of ghrelin in ovariectomized (OVX) obese rats fed with a high-fat diet (HFD). Four weeks after OVX at 9 weeks of age, Wistar rats were subcutaneously implanted with either 17β-estradiol (E2) or placebo (Pla) pellets and started on HFD feeding. After 4 weeks, growth hormone-releasing peptide (GHRP)-6, a growth hormone secretagogue receptor (GHSR) agonist injected intraperitoneally, induced changes in HFD intake, and c-Fos-positive neurons in the hypothalamic arcuate nucleus (ARC) were measured in both groups. The ghrelin protein and mRNA levels, as well as GHSR protein in stomach, were analyzed by Western blotting and real-time PCR. HFD increased energy intake and body weight in the Pla group, while it temporarily reduced these in the E2 group. GHRP-6 enhanced HFD intake and activated neurons in the ARC only in the Pla group. Furthermore, gastric ghrelin and GHSR protein levels were lower in the E2 group than in the Pla group, but plasma acyl ghrelin levels were similar in both groups. Our results suggest that E2 replacement improves obesity by inhibiting the orexigenic action of ghrelin via downregulation of ghrelin and its receptor in stomach in HFD-fed OVX rats.

## 1. Introduction

Obesity, defined as excessive fat accumulation, has become a worldwide epidemic [1]. Overweight and fat accumulation are associated with many adverse health outcomes, including type 2 diabetes, stroke, and heart disease [2]. Some studies have shown that dietary composition, particularly high-fat diet (HFD), promotes obesity in humans [3] and mice [4]. Furthermore, ovarian hormones play an important role in the control of food intake and body weight in female mammals [5,6]. The onset of menopause is accompanied by increases in energy intake and appetite and a decrease in energy consumption, followed by an increase in total body fat and visceral adipose tissue mass [7,8]. Hormone replacement therapy (HRT) attenuates body weight gain and fat accumulation in postmenopausal women [9,10]. However, to our knowledge, the effect of menopause or HRT in women consuming HFD remains unclear. Moreover, previous studies using ovariectomized (OVX) rats, an animal model widely used for studying the pathology of human menopause, showed that 17β-estradiol (E2) replacement suppresses body weight gain and fat accumulation and enhanced whole-body energy consumption not only under a normal standard diet (SD) [11,12] but also under HFD [13]. HFD-induced obesity in OVX rats shares several features with diet-related obesity in postmenopausal women. It is plausible that E2 replacement improves obesity through its regulatory effects on food intake, even under conditions where HFD is easily accessible. However, the mechanism underlying the anorexigenic effect of E2 replacement on HFD intake is yet to be clarified.

Recently, several studies have reported that the regulatory effects of E2 on food intake, energy balance, and fat accumulation are mediated through its interaction with orexigenic and anorexigenic hormones [14,15]. Ghrelin is a candidate whose orexigenic action is regulated by E2 [16]. Ghrelin, a brain-gut peptide hormone secreted from the stomach, stimulates food intake via the action on its receptor, the growth hormone secretagogue receptor (GHSR) [17,18]. Ghrelin is mainly synthesized in X/A-like endocrine cells of the gastric oxyntic mucosa [19]. GHSR is produced in the neurons of the vagus nerve ganglion and transported to nerve endings in the stomach, where it binds to ghrelin [20,21]. Orexigenic signals from ghrelin are transmitted to the brain via either a neural or a hormonal pathway, or through both [22]. The neural pathway consists of afferent vagal fibers that pass through the nodose ganglia and terminate in the nucleus tractus solitarius located in the brainstem to transmit their signals ultimately to the hypothalamus. A previous study suggested that the vagal afferent nerve-dependent pathway was involved in peripherally injected ghrelin-induced increase in food intake and neural activation in the hypothalamic arcuate nucleus (ARC), a key center regulating food intake and satiety [20]. ARC contains orexigenic neurons expressing the neuropeptide Y (NPY) and agouti-related protein (AgRP) and anorexigenic neurons expressing proopiomelanocortin (POMC) and cocaine- and amphetamine-regulated transcript (CART) [23]. Peripheral ghrelin increases the number of c-Fos-positive neurons in the NPY/AgRP neurons in the ARC in rodents [24,25]. Because intranuclear c-Fos is the product of the immediate-early-gene c-fos, an established marker of changes in neuronal activity in response to stimuli in rodents [26,27], these findings indicate that ghrelin activates orexigenic neurons in ARC, resulting in an increase in food intake. The hormonal pathway also may reach the hypothalamus directly via the blood circulation, but details of the route remain unclear.

A recent study reported that female GHSR-null mice accumulated less food intake, body weight, and adiposity than wild-type mice when given HFD [28]. Additionally, OVX increased food intake and body weight in wild-type female mice, but these effects were not observed in the GHSR-null mice [16]. A previous study also reported that 7-days subcutaneous treatment with a GHSR antagonist, [D-Lys^3^]-growth hormone-releasing peptide (GHRP)-6, decreased food intake in OVX mice fed with either HFD or SD, and the antagonistic effect was reduced after E2 supplementation [29]. Taken together, GHSR might be involved in the inhibitory effects of E2 on food intake and body weight gain. In contrast, it has been reported that a change in plasma estrogen level during estrous cycle had no effects on GHSR mRNA expression at least in the ARC of intact female rats, despite the fact that the orexigenic effect and the c-Fos-inducing effect of intracerebroventricular ghrelin were influenced by the estrous cycle phase [30]. Therefore, the mechanism accounting for the anorexigenic effects of E2 associated with GHSR or the action site of E2 regulating GHSR remains unclear. We have focused on the effects of E2 on ghrelin action via GHSR in the stomach, especially under HFD feeding in OVX rats. Most of the previous studies, which demonstrated the inhibitory effects of estrogen on ghrelin’s eating-stimulatory action, were performed using SD-fed rodents. To our knowledge, no previous report has investigated the mechanisms accounting for the inhibitory effect of E2 replacement on HFD-induced obesity in OVX rats.

The aim of this study is to determine whether chronic E2 replacement in physiological dosage attenuates HFD intake by suppressing the orexigenic action of ghrelin through the ghrelin-GHSR-ARC pathway in HFD-fed OVX rats. Moreover, we examined the effects of E2 replacement on intraperitoneal (IP) GHSR agonist, GHRP-6-induced orexigenic response, and neural activation in ARC, on plasma acyl ghrelin concentrations, and on ghrelin protein, ghrelin mRNA, and GHSR protein levels in the stomach in order to clarify the underlying mechanism of estrogen action in OVX rats.

## 2. Materials and Methods

### 2.1. Animals and Diets

Animal procedures were approved by The Nara Women’s University Committee on Animal Experiments (No. 17-02) and were conducted in accordance with the Standards relating to the Care and Keeping and Reducing Pain of Laboratory Animals (Notice of the Ministry of the Environment, Government of Japan) and ARRIVE guidelines. In total, 88 female Wistar rats (CLEA Japan, Inc., Tokyo, Japan) were used in this study. The rats were individually housed in standard polycarbonate cages containing paper bedding under controlled temperature and light conditions (26 ± 1 °C, a 12:12-h light–dark cycle, with lights on at 7:00 AM). Tap water and rodent chow were provided ad libitum. All surgeries were performed while the rats were under anesthesia (pentobarbital sodium; 25–40 mg/kg IP or isoflurane; 1.5–2.0% in oxygen).

Nine-week-old female rats fed on SD (MF; Oriental Yeast, Tokyo, Japan) were ovariectomized followed by E2 or placebo (Pla) replacement as previously described [12,31]. In brief, after a 4-week recovery period, the rats were assigned randomly to either the Pla (*n* = 48)- or the E2 (*n* = 40)-treated group and were subcutaneously implanted with either E2 (1.5 mg/60-day release) or Pla pellets (Innovative Research of America, Sarasota, FL, USA). Additionally, HFD (F2HFD2; Oriental Yeast) containing 515.7 kcal per 100 g (60.7% energy from fat, predominantly lard) was started the day after the replacement and was continued until the cessation of the experiments for HFD-fed rats in both groups. The SD-fed rats continued to receive SD, which contained 360.0 kcal per 100 g (13.2% energy from fat). Food intake and body weight were measured daily during the experiments.

### 2.2. Experimental Protocols

After 4 weeks of replacement therapy, the subsets of 17-week-old rats were used for IP injection of GHRP-6 (Sigma-Aldrich, St. Louis, MO, USA) (Pla, *n* = 13; E2, *n* = 14). One week after the experiment, the rats were used in immunohistochemical analysis (Pla, *n* = 9; E2, *n* = 9). A separate set of 17-week-old rats was used for blood sampling for measurements of plasma acyl ghrelin and for stomach sampling for ghrelin protein, ghrelin mRNA, and GHSR protein analyses (Pla, *n* = 9–11; E2, *n* = 8–10). Furthermore, the wet weights of the intra-abdominal (mesenteric, kidney–genital, and retroperitoneal) and subcutaneous (inguinal) adipose tissues were measured (Pla, *n* = 34; E2, *n* = 26).

### 2.3. IP Injection of GHRP-6

The experiment to examine the effects of GHRP-6 was performed 4 weeks after the replacement using satiated rats with free access to HFD. HFD intake was measured hly from 2 h before and up to 4 h after GHRP-6 injection. The rats were injected intraperitoneally either with GHRP-6 dissolved in saline (80 nmol/kg or 400 nmol/kg) or saline at 11:00 AM. The animals had free access to water during the experiments.

### 2.4. Immunohistochemistry

The rats aged 18 weeks were injected intraperitoneally either with GHRP-6 dissolved in saline (400 nmol/kg) or saline followed by 3-h fasting. At 3 h after the injection, the rats were deeply anesthetized with sodium pentobarbitone (45 mg/kg, IP) and transcardially perfused with ice-cold phosphate-buffered saline (PBS), followed by 4% paraformaldehyde in a 0.1 M-phosphate buffer (pH 7.4) for fixation. The removed brains were post-fixed in fixative at 4 °C and then immersed in PBS containing 15% sucrose for 1 day, followed by 25% sucrose for 2 days for cryoprotection. Then, 30-µm coronal sections of the brain were prepared using a cryostat (Leica CM3050 S; Leica Biosystems, Wetzlar, Germany). c-Fos immunohistochemical staining in the ARC was performed using a rabbit c-Fos antibody (dilution 1:4000, Sc52; Santa Cruz Biotechnology, Dallas, TX, USA) as previously described [32]. The sections were incubated in biotinylated goat anti-rabbit IgG (dilution 1:400, BA-100; Vector Laboratories, Burlingame, CA, USA), followed by ABC Elite Kit solution (dilution 1:400, Vector Laboratories). Visualization of the antibodies was performed using 0.02% 3,3-diaminobenzidine (Dojindo Laboratories, Kumamoto, Japan) and 0.01% H_2_O_2_ in 50 mM Tris HCl buffer (pH 7.4). The sections were mounted on gelatin-coated glass slides, dehydrated with graded ethanol, cleared with Lemosol^®^, and coverslipped. Four consecutive sections containing ARC (in between −2.16 to −2.28 mm from the bregma) were observed using a microscope (Olympus BX-50; Olympus Corporation, Tokyo, Japan), and images were obtained using a cooled charge-coupled device camera (Micropublisher RTV 5.0; QImaging, Surrey, BC, Canada). The ARC region was identified using the rat brain stereotaxic atlas [33]. The number of c-Fos-positive neurons in the ARC was counted for each of the four consecutive sections using image analysis software (ImageJ; NIH, Bethesda, MD, USA). The results were expressed as the average number of c-Fos-positive neurons per bilateral sections in the ARC.

### 2.5. Sampling of Gastric Mucosae and Measurement of Adipose Tissues

After 16 h of fasting, a subset of Pla and E2 rats were euthanized by a pentobarbital sodium overdose. Mucosa from upper and middle portions of the stomach was harvested between 10:00 a.m. and 2:00 p.m. The mucosae for GHSR and ghrelin protein analysis were immediately frozen in liquid nitrogen before being stored at −50 °C until further processing. The tissues were stored in RNA stabilization solution until RT-qPCR analysis for ghrelin was performed. Upon completion of the experiments, the rats were sacrificed by a pentobarbital sodium overdose, and wet weights of the intra-abdominal (mesenteric, kidney–genital, and retroperitoneal) and subcutaneous (inguinal) adipose tissues were measured. The total weight of the visceral adipose tissues was calculated as the sum of the intra-abdominal fat weights.

### 2.6. Immunoblotting

Isolated gastric mucosae were immediately homogenized in homogenization buffer, as previously described [12]. The homogenates were centrifuged at 15,000× *g* for 30 min at 4 °C. Sodium dodecyl sulfate (SDS) samples containing equal amounts of protein were separated by SDS-polyacrylamide gel electrophoresis on 10% polyacrylamide gels and immunoblotted using a PVDF membrane (GE Healthcare Life Sciences, Buckinghamshire, UK) with the following antibodies: Antibodies for ghrelin (1:2000) and the β-actin (1:2000) from Sigma-Aldrich, and GHSR (1:1000) from Abcam (Cambridge, MA, USA). The primary antibodies were detected with anti-rabbit (Promega, Madison, WI, USA) or anti-chicken (Abcam) IgG horseradish peroxidase-conjugated secondary antibody. An enhanced chemiluminescence (ECL; GE Healthcare Life Sciences) system was used for protein detection. Imaging and densitometry were performed using the Ez-Capture imaging system (ATTO, Tokyo, Japan) and the CS Analyzer image processing program (ATTO).

### 2.7. RNA Isolation and RT-qPCR

Total RNA was extracted using the TRI Reagent Solution (Ambion, Austin, TX, USA) according to the manufacturer’s protocol. The amount of total RNA extracted was determined, and its purity (absorption ratio of optical density at 260 and 280 nm >1.9) was verified spectrophotometrically using a Nanodrop 2000 (Thermo Fisher Scientific, Wilmington, DE, USA). Then, the cDNA was synthesized using the High-Capacity RNA-to-cDNA kit (Applied Biosystems, Carlsbad, CA, USA). RT-qPCR was performed using a StepOne Software v2.1 system (Applied Biosystems). The commercially available TaqMan Gene Expression Assay (Applied Biosystems) for ghrelin (Rn00572319_m1) and β-actin (Rn00667869_m1) was used in this study. For the analysis, gene expression levels of ghrelin were normalized using β-actin as a housekeeping gene and expressed with respect to the average value for the Pla group. All reactions were performed in duplicate. The thermal cycling conditions were as follows: 95 °C for 20 s, followed by 40 cycles at 95 °C for 1 second and 60 °C for 20 s. No amplification of fragments occurred in the control samples without reverse transcriptase. The mRNA quantity was calculated using the ΔΔCt (comparative Ct) method under the assumption that the primer efficiencies were relatively similar.

### 2.8. Assessment of Plasma Acyl Ghrelin Level

Freely moving rats aged 16 weeks in separate sets of Pla (*n* = 9) and E2 groups (*n* = 8) were catheterized with venous cannula, as previously described [34]. Blood sampling was performed on the 5th-day post-cannulation. After 16 h of fasting and 1 h of rest after connecting the cannula with the extension tube that allowed free movement, a blood sample (0.2 ml) was collected from the cannula between 11:00 a.m. and 1:00 p.m. and harvested in plastic tubes containing 50 mg/ml ethylenediamine tetraacetic acid. The plasma was immediately treated with 1/10 volume of 1 mol/L HCl and stored at 45 °C until assayed for acyl ghrelin using an active ghrelin ELISA kit that recognizes n-octanoylated ghrelin (Mitsubishi Chemical Medience Corporation, Tokyo, Japan).

### 2.9. Statistical Analyses

All values are expressed as means ± standard error (SE). Two-way repeated measures ANOVA for each pair-wise comparison among the total four (Pla–SD, Pla–HFD, E2–SD, and E2–HFD) groups, followed by a Tukey–Kramer post hoc test was used to analyze the effect of E2 or HFD on the course of change in food intake, energy intake, body weight, and the effect of E2 or GHRP-6 on the course of change in HFD and cumulative HFD intakes. Two-way factorial ANOVA followed by a Tukey-Kramer post hoc test was used to analyze the effect of E2 on HFD-induced change in wet weights of adipose tissues and the effect of E2 on GHRP-6-induced change in the number of c-Fos-positive neurons between the groups. The ghrelin, GHSR proteins, and ghrelin mRNA levels in the stomach, and plasma acyl ghrelin between the Pla and E2 (HFD-fed rats) groups were compared using unpaired *t*-test. We considered a value of *p* < 0.05 to be statistically significant.

## 3. Results

### 3.1. Characterization of the Studied Rats

**Figure 1:**
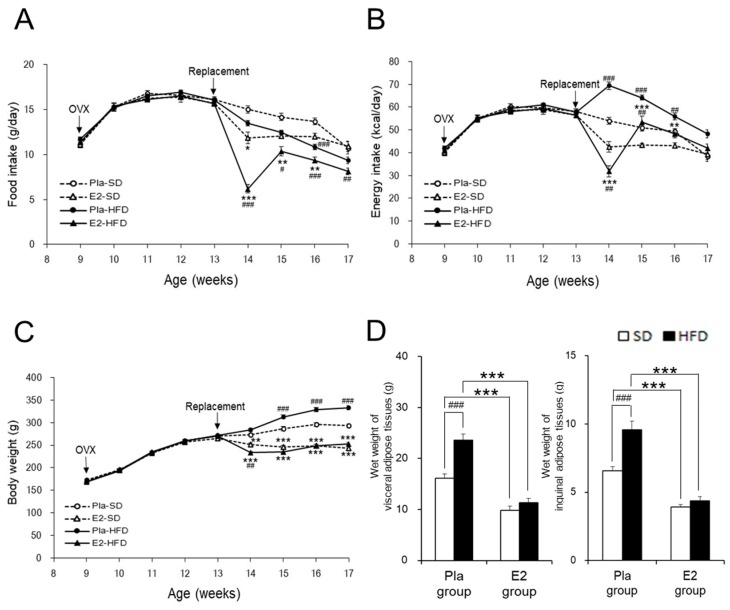
Characterization of the studied rats. Data are expressed as means ± SE. Line graphs represent the course of change in mean food intake (**A**), energy intake (**B**), and body weight (**C**) in the placebo (Pla)-standard diet (SD) (*n* = 18), 17β-estradiol (E2)-SD (*n* = 14), Pla-high-fat diet (HFD) (*n* = 30), and E2-HFD (*n* = 26) groups of ovariectomized (OVX) rats. Two-way repeated-measures ANOVA for each pair-wise comparison among the four groups revealed significant differences in food intake, energy intake, and body weight. There was an interaction of age and group effects in food intake (*p*_Age × Group_ < 0.001: E2–HFD vs. E2–SD or Pla–HFD), energy intake (*p*_Age × Group_ < 0.001: Pla–HFD vs. Pla–SD or E2–HFD) and body weight (*p*_Age × Group_ < 0.05: Pla–SD vs. Pla–HFD, *p*_Age × Group_ < 0.01: Pla–SD vs. E2–SD, and *p*_Age × Group_ < 0.001: Pla–HFD vs. E2–HFD). * *p* < 0.05, ** *p* < 0.01, and *** *p* < 0.001, differences between the E2 and Pla groups; ^#^ *p* < 0.05, ^##^ *p* < 0.01, and ^###^ *p* < 0.001, differences between the SD and HFD groups. Bar graphs represent wet weights of visceral (the sum of the mesenteric, kidney–genital, and retroperitoneal adipose tissue weights) and inguinal adipose tissues (**D**) at 17 weeks of age in the Pla–SD (*n* = 18), the Pla–HFD (*n* = 16), the E2–SD (*n* = 14), and the E2–HFD (*n* = 12) groups. Data were analyzed by two-way factorial ANOVA, followed by a post hoc Tukey–Kramer test. There was an interaction of diet and group effects in the wet weights of visceral (*p*_Diet × Group_ < 0.01) and inguinal adipose tissues (*p*_Diet × Group_ < 0.01). *** *p* < 0.001, differences between the E2 and Pla groups; ^###^ *p* < 0.001, differences between the SD and HFD groups.

Regardless of diet, food intake remarkably decreased in the E2 group compared with the Pla group at 14 weeks of age one week after the E2 replacement and returned to control levels as indicated by the Pla group at 17 weeks of age (Figure 1A). HFD decreased food intake between 14–17 weeks of age, compared with SD, only in the E2 group. In contrast, HFD increased energy intake between 14–16 weeks of age, compared with SD, in the Pla group. Therefore, the energy intake from HFD during this period was significantly higher in the Pla group compared to the E2 group, but not in the two SD-fed groups (Figure 1B).

HFD increased body weight compared with SD in the Pla group between 15–17 weeks of age, but not in the E2 group, resulting in the difference between the two groups (Figure 1C). The wet weights of total visceral and inguinal subcutaneous adipose tissues (Figure 1D) were significantly increased by HFD in the Pla group, but not in the E2 group. Taken together, E2 replacement suppressed body weight gains and fat accumulation by adjusting the energy intake.

### 3.2. Effect of the IP GHRP-6 Injection on HFD Intake

**Figure 2:**
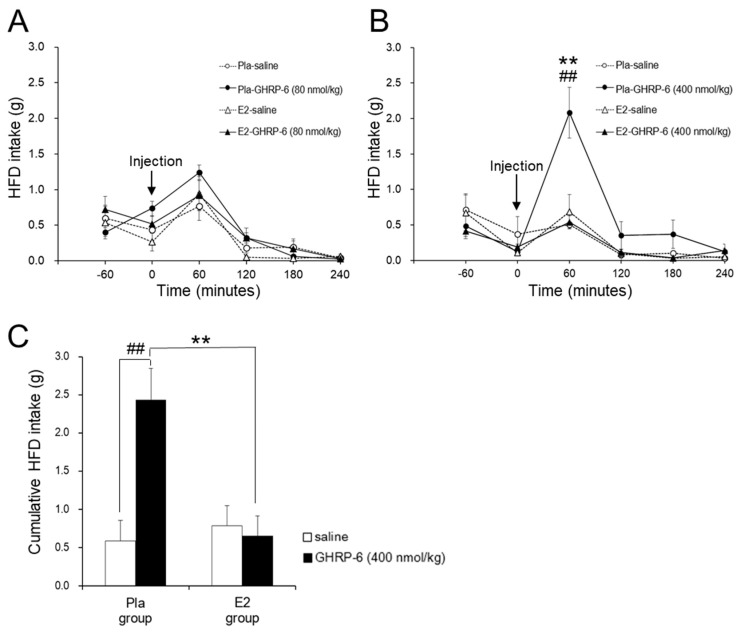
Effect of the intraperitoneal (IP) growth hormone-releasing peptide (GHRP)-6 injection on high-fat diet (HFD) intake. Line graphs represent changes in HFD intake after IP injection of GHRP-6 (**A**: 80 nmol/kg, **B**: 400 nmol/kg) or saline in the placebo (Pla; *n* = 13), 17β-estradiol (E2; *n* = 14) groups of ovariectomized HFD-fed rats. Data are expressed as means ± SE and were analyzed by two-way repeated-measures ANOVA for each pair-wise comparison among the four groups. There was an interaction of time and group effects in the HFD intake after GHRP-6 injection at a dose of 400 nmol/kg (*p*_Time × Group_ < 0.05: Pla–GHRP-6 vs. Pla–saline or E2–GHRP-6). Significant differences were observed at 60 min after GHRP-6 injection at a dose of 400 nmol/kg between the Pla–GHRP-6 and the E2–GHRP-6 groups (** *p* < 0.01), and between the Pla–saline and the Pla–GHRP-6 groups (^##^ *p* < 0.01). Bar graphs represent the cumulative HFD intake for 2 h after GHRP-6 injection at a dose of 400 nmol/kg in the Pla and E2 groups (**C**). Two-way repeated-measures ANOVA showed an interaction of injection and group effects in the HFD intake (*p*_Injection × Group_ < 0.01). Significant differences in HFD intakes were observed between the Pla–saline and the Pla–GHRP groups (^##^ *p* < 0.01), and between the Pla–GHRP-6 and the E2–GHRP-6 groups (** *p* < 0.01).

GHRP-6 at a dose of 80 nmol/kg had no effect on HFD intake in the Pla and E2 groups (Figure 2A). However, GHRP-6 at a dose of 400 nmol/kg enhanced HFD intake at 60 min after the injection compared with saline only in the Pla group but not in the E2 group (*p* < 0.01) (Figure 2B). Figure 2C shows that IP injection of GHRP-6 at a dose of 400 nmol/kg enhanced cumulative HFD intake for 120 min after the injection only in the Pla group (*p* < 0.01) but not in the E2 group, resulting in a difference in the GHRP-6-induced response between the two groups (interaction, *p* < 0.01).

### 3.3. In Vivo Effects of E2 Replacement on Ghrelin and GHSR in the Stomach

**Figure 3:**
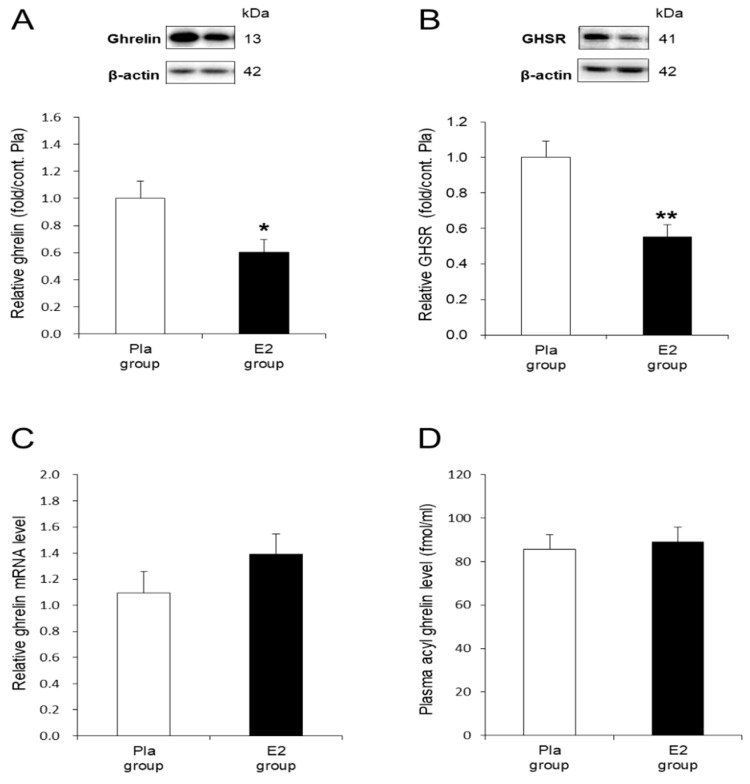
In vivo effects of 17β-estradiol (E2) replacement on ghrelin and growth hormone secretagogue receptor (GHSR) in the stomach. (**A**) Representative blots and relative values of ghrelin protein level in the gastric mucosa of HFD-fed rats in the placebo (Pla, *n* = 9)- and E2 (*n* = 8)-treated groups. (**B**) Representative blots and relative values of GHSR protein level in the stomach of the rats in the Pla (*n* = 11) and E2 (*n* = 10) groups. (**C**) Relative values of ghrelin mRNA level in the gastric mucosa of the rats in the Pla (*n* = 8) and the E2 (*n* = 8) groups. Data are evaluated as 2^−ΔΔCt^ using β-actin as a housekeeping gene. (**D**) Plasma acyl ghrelin level after 16-h fasting in the Pla (*n* = 9) and the E2 (*n* = 8) groups at 17 weeks of age. Data are expressed as means ± SE and were analyzed by unpaired *t*-test. * *p* < 0.05 and ** *p* < 0.01, differences between E2 and Pla groups.

To examine the molecular mechanism accounting for the inhibitory effect of E2 on the orexigenic action of ghrelin in the OVX rats, we investigated ghrelin and GHSR protein levels in the gastric mucosa. The E2 group showed significantly suppressed ghrelin (*p* < 0.05) and GHSR (*p* < 0.01) protein levels compared with the Pla group (Figure 3A,B). In contrast, the levels of ghrelin mRNA in the gastric mucosa were not different between the two groups (Figure 3C). As shown in Figure 3D, the plasma acyl ghrelin concentrations after 16 h of fasting at 17-weeks-old were similar between the two groups.

### 3.4. c-Fos-Positive Neurons in the ARC Induced by IP Injection of GHRP-6

**Figure 4:**
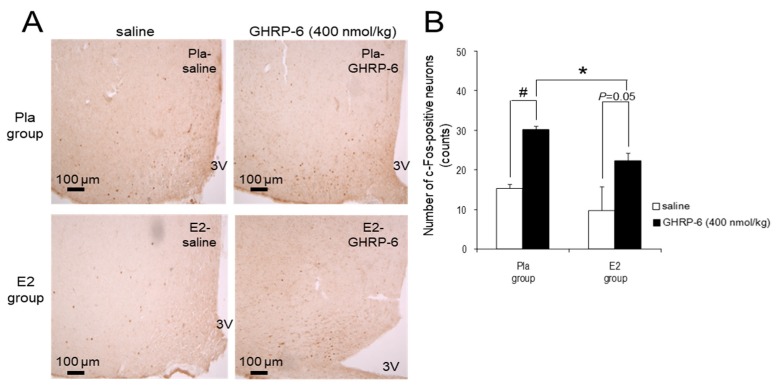
c-Fos-positive neurons in the arcuate nucleus (ARC) induced by intraperitoneal (IP) injection of growth hormone-releasing peptide (GHRP)-6. (**A**) Representative images of c-Fos-positive neurons after IP injection of 400 nmol/kg GHRP-6 or saline in the ARC of high-fat diet (HFD)-fed rats in the placebo (Pla)-saline, Pla–GHRP-6, 17β-estradiol (E2)–saline, and E2–GHRP-6 groups. 3V, third ventricle. Scale bars: 100 μm. (**B**) The number of the c-Fos-positive neurons in the ARC after IP injection of 400 nmol/kg GHRP-6 or saline in the Pla–saline (*n* = 4), Pla–GHRP-6 (*n* = 5), E2–saline (*n* = 4) and E2–GHRP-6 (*n* = 5) groups. Values are shown as the mean ± SE. Data were analyzed by two-way factorial ANOVA, followed by a post hoc Tukey–Kramer test. * *p* < 0.05, differences between the E2 and Pla groups. ^#^ *p* < 0.05, differences between the GHRP-6 and saline groups.

The injection of GHRP-6 at a dose of 400 nmol/kg enhanced the number of the c-Fos-positive neurons in the ARC in the Pla group compared with the injection of saline (*p* < 0.05), but it did not reach significance (*p* = 0.05) in the E2 group. Furthermore, the number of c-Fos-positive neurons after the injection was significantly lower in the E2 group compared with the Pla group (*p* < 0.05; Figure 4).

## 4. Discussion

The present study shows that E2 replacement reduces energy intake from HFD by suppressing orexigenic effects of ghrelin via downregulations of ghrelin and GHSR proteins levels in the stomach of HFD-fed OVX rats.

In this study, E2 replacement suppressed the increase in energy intake and body weight gain in HFD-fed OVX rats much more than in SD-fed rats. To our knowledge, our study is the first to show strongly suppressive effects of E2 replacement on HFD-induced energy intake increase and obesity compared with SD-fed OVX rats. On the contrary, HFD-fed OVX mice, despite being more obese, showed slightly lower food intake than the E2-replaced mice [35] or showed similar body weight to the E2-replaced mice under 1-month HFD feeding [36]. These discrepancies may be attributed to the differences in species between mice and rats. In our previous study, we showed that OVX increased food intake and intra-abdominal fat accumulation, which were improved by E2 replacement in the SD-fed OVX rats [12]. In addition, Witte et al. reported that OVX led to hyperphagia in rats but did not in mice and that OVX-induced weight gain in female mice was mediated by reduced locomotor activity and metabolic rate [37]. Therefore, we used rats as experimental animals to investigate the effects of E2 on food intake via ghrelin action.

IP injection of GHRP-6 at a dose of 400 nmol/kg increased the cumulative HFD intake for 120 min by approximately 2.5 times and enhanced the number of c-Fos-positive neurons in the ARC by about two times compared with saline injection in OVX rats. The dose is comparable to one-third of the daily dosage in a previous study showing that the chronic GHRP-6 infusion by a pump increased food intake in male rats [38]. Additionally, the administration of 3 nmol/rat (250–300 g) ghrelin (IP) increased food intake threefold [39] and the number of c-Fos-positive neurons in the ARC twofold compared with saline in male rats [40]. Therefore, a GHRP-6 dose of 400 nmol/kg may induce a comparable response in OVX rats to approximately 10 nmol/kg ghrelin in male rats. In contrast, a GHRP-6 dose of 80 nmol/kg had no effect on food intake in both groups. This dose is likely insufficient to produce an orexigenic neural activation in the ARC. In addition, the 400 nmol/kg dose-induced increase of food intake in the OVX group was inhibited completely in the E2 group. Therefore, we used only the 400 nmol/kg dose of GHRP-6 for the immunohistochemical analysis to evaluate the neural activation indicated by the increased number of c-Fos-positive neurons in the ARC, which regulate food intake.

Our results suggest that E2 replacement may regulate GHSR protein in the stomach in OVX rats. A recent study using GHSR-null mice reported that the abundance of GHSR-expressing neurons in the nodose ganglion is critical for peripheral ghrelin administration-induced hyperphagia via the vagus nerve [41]. A presumable target site of E2 may be the cell bodies of the vagal nodose ganglion, but no report has investigated the relationship between E2 and GHSR. Clegg et al. [16] showed that the increases in feeding and body weight in wild-type OVX mice were blunted in GHSR-null mice. This finding suggests a pivotal role of GHSR in OVX-related body weight gain, though the target site of ovarian hormones remains unclear. The present study demonstrates for the first time that E2 replacement suppresses energy intake and obesity via the reduction of GHSR protein level in the stomach of HFD-fed OVX rats. A previous report has shown that the estrogen receptor (ER) exists in vagal nodose ganglionic neurons of the rat [42]. Therefore, estrogen may have affected GHSR gene expression through ER in the vagal afferent neurons. Further studies are required to elucidate whether the ERs co-localize with GHSR in the nodose ganglionic neurons of vagal afferent nerve and downregulate GHSR mRNA followed by reduction of GHSR protein level in the stomach. Additionally, we consider the possibility that the reduction of GHSR level in the stomach of E2-replaced rats is a secondary effect caused by the decreased gastric ghrelin levels observed in this study.

Previous studies have suggested that gastric vagal afferent is the major pathway involved in peripherally injected ghrelin-induced increase in food intake and c-Fos immunoreactivity in the ARC [20,43]. Most of the ghrelin actions in ARC are realized via the NPY/AgRP neurons pathway [18], and simultaneously, ghrelin suppresses anorexigenic POMC/CART neurons [44]. The present study has shown that E2 replacement suppressed IP GHRP-6-induced HFD intake and simultaneous neuronal activation in the ARC of OVX rats. It is plausible that the c-Fos-positive cells in which the numbers were increased by GHRP-6 in ARC are orexigenic NPY/AgRP neurons. However, further studies are necessary to clarify whether c-Fos-positive cells, which were downregulated by E2 replacement in HFD-fed OVX rats, are NPY/AgRP neurons or others such as tyrosine hydroxylase neurons [45].

Interestingly, E2 replacement reduced ghrelin protein levels in the stomach of OVX rats in the present study. This finding is likely related to the mechanism of suppressive effects of E2 replacement on HFD intake in OVX rats. In contrast, E2 replacement had no effect on the ghrelin mRNA in the stomach of OVX rats. Consequently, these results suggest that the reduction of ghrelin protein is mediated by a post-transcriptional mechanism. In previous studies, Northern blot analysis showed that ghrelin mRNA expression in the stomach increased 3 days after OVX in female rats aged 4 weeks, but not in rats aged 9 weeks [46] and that it was not affected by OVX in adult rats [47]. Another study reported that E2 replacement had no effects on gastric ghrelin mRNA levels in the 4-week-old OVX rats, although OVX increased gastric ghrelin secretion in female rats, which was restored by E2 replacement [48]. Therefore, our results are consistent with these findings, suggesting no effects of OVX or E2 on gastric ghrelin mRNA at least in adult female rats, although the animal models used were different. To our knowledge, this is the first data to show that E2 replacement attenuates ghrelin protein levels in the stomach of the postmenopausal model rats.

The other route for conveying the gastric-derived ghrelin signal to the brain is the blood circulation [22]. Our results suggest that the E2 replacement had no effect on the plasma acyl ghrelin level, although it decreased gastric ghrelin proteins in OVX rats. Whereas, ghrelin is widely expressed in peripheral tissues such as the intestine and pancreas [22] in addition to the stomach, plasma ghrelin might not depend on the gastric ghrelin level as shown in our result. In addition, the proportion of acyl ghrelin to that of total ghrelin has been reported to be far smaller proportion at 2–5% in the plasma than that at 20–40% in the stomach in rodents [49,50]. These findings may be reasons why E2 had an inhibitory effect on the gastric ghrelin protein, but not on plasma acyl ghrelin in this study. Moreover, Cleggs et al. [16] found that plasma ghrelin and food intake were transiently increased 2 to 4 weeks after OVX in SD-fed rats. Another study in female rats showed that the plasma ghrelin level increased 3 days after OVX but returned to the basal level 7 days after OVX in 4-week-old rats [46]. Our results showing that 4-week E2 replacement to rats 4 weeks after OVX had no effect on plasma ghrelin level are consistent with these findings. This may be another reason for no clear effects of E2 on plasma acyl ghrelin in this study.

Furthermore, we have to take into account the possibility that the inhibitory effects of E2 on ghrelin action are intermediated by other peptides regulating food intake such as leptin because a conditional deletion of leptin receptor from the vagal afferent neurons failed to protect exogenous ghrelin-induced hyperphagia in female mice [51].

In this study, the rats in the E2 group are considered postmenopausal models replaced with E2 because they were used for the experiment 4 weeks after E2 replacement in a 4-week post-OVX. In addition, the plasma E2 concentration in the E2 group might be within the accepted physiological range, as shown in our previous study (44.1 ± 10.3 and 9.2 ± 0.5 pg/ml in the E2 and Pla groups, respectively) using E2 pellets containing the same dosage as in the present study [12]. Therefore, our results provide the animal data to support that E2 replacement reverses the postmenopausal obesity exacerbated by HFD. Menopause was associated with an increase in total body fat and visceral abdominal fat [7]. The percentage of energy intake from fat significantly increased in the postmenopausal years, though the decrease in this variable was observed during the menopausal transition [8]. Our results suggest that postmenopausal women need to avoid a HFD and control dietary fats appropriately to prevent obesity.

## 5. Conclusions

In conclusion, the present study suggests that E2 replacement reverses the HFD-induced increase in energy intake and fat accumulation by suppressing orexigenic ghrelin action via the reduction of GHSR and ghrelin in the stomach of OVX rats. Our findings may provide insight into the mechanism of diet-induced postmenopausal obesity as well as the beneficial effects of HRT against obesity in postmenopausal women. Ghrelin and GHSR in the stomach may be new targets for strategies in medicines and dietary habits to improve postmenopausal obesity.
